# Preparation and Evaluation of Eudragit L100-PEG Proliponiosomes for Enhanced Oral Delivery of Celecoxib

**DOI:** 10.3390/pharmaceutics12080718

**Published:** 2020-07-30

**Authors:** Min-Hwan Kim, Dong Hyun Kim, Duy-Thuc Nguyen, Han Sol Lee, Nae-Won Kang, Min-Jun Baek, Jiseon An, So-Yeol Yoo, Yong-Hyeon Mun, Wonhwa Lee, Ki-Taek Kim, Cheong-Weon Cho, Jae-Young Lee, Dae-Duk Kim

**Affiliations:** 1 College of Pharmacy and Research Institute of Pharmaceutical Sciences, Seoul National University, Seoul 08826, Korea; mhkim305@naver.com (M.-H.K.); nguyenduythuc92@snu.ac.kr (D.-T.N.); nwkangkr@naver.com (N.-W.K.); bmybook@snu.ac.kr (M.-J.B.); 2 College of Pharmacy, Chungnam National University, Daejeon 34134, Korea; gcy70426@gmail.com (D.H.K.); sol4273@naver.com (H.S.L.); anthony0213@kakao.com (J.A.); nature4857@naver.com (S.-Y.Y.); ansdydtus@gmail.com (Y.-H.M.); chocw@cnu.ac.kr (C.-W.C.); 3 Aging Research Center, Korea Research Institute of Bioscience and Biotechnology, Daejeon 34141, Korea; wonhwalee@kribb.re.kr; 4 College of Pharmacy and Natural Medicine Research Institute, Mokpo National University, Jeonnam 58554, Korea; ktkim0628@mokpo.ac.kr

**Keywords:** proliponiosomes, Eudragit L100, PEGylation, celecoxib, oral bioavailability

## Abstract

PEGylated Eudragit L100 (ELP)-containing proliponiosomes (PLNs) were developed for improved oral delivery of celecoxib (CXB). The successful introduction of PEG 2000 or 5000 to Eudragit L100 (EL) was confirmed via proton nuclear magnetic resonance analysis of which calculated molar substitution ratio of PEG to EL was 36.0 or 36.7, respectively. CXB, ELP, phospholipid, and non-ionic surfactants were dissolved in dimethyl sulfoxide and lyophilized to produce CXB-loaded PLNs (CXB@PLNs). The physical state of CXB@PLNs was evaluated using differential scanning calorimetry and powder X-ray diffractometry, which revealed that crystalline CXB was transformed into amorphous form after the fabrication procedure. The reconstitution of CXB@PLNs in aqueous media generated CXB-loaded liponiosomes with nano-sized mean diameters and spherical morphology. CXB@PLNs displayed enhanced dissolution rate and permeability compared to CXB suspension. In vivo pharmacokinetic studies performed on rats demonstrated the improved oral bioavailability of CXB@PLNs compared to that of CXB suspension. No serious systemic toxicity was observed in the blood biochemistry tests performed on rats. These results suggest that the developed PLNs could be promising oral delivery systems for improving the bioavailability of poorly water-soluble drugs, such as CXB.

## 1. Introduction

Celecoxib (CXB) is a nonsteroidal anti-inflammatory drug (NSAID) indicated for osteoarthritis, rheumatoid arthritis, ankylosing spondylitis, acute pain, primary dysmenorrhea, and familial adenomatous polyposis [1,2]. Given the selectivity to cyclooxygenase (COX)-2, CXB provides higher efficacy with less gastrointestinal (GI) side effects compared to non-selective COX inhibitors [3]. However, its poor solubility in aqueous media (1.4 and 1.2 μg/mL at pH 1.2 and 6.8, respectively) causes limited and highly variable oral absorption [2], which compromises its therapeutic efficacy [4]. To address this issue, Celebrex, a commercially available CXB product, contains sodium dodecyl sulfate (SDS) as a solubilizer. Although this anionic surfactant is well-known for its high drug solubilizing potential with the characteristic micelle-forming property [5], several toxicity concerns have always been disputed, such as skin and mucosal irritations [6]. Thus, various formulation approaches have been made to improve the solubility and dissolution rate of CXB without using anionic surfactants. These strategies can be categorized into mainly two types: amorphization [6,7,8] and nanonization [9,10,11].

The generation and maintenance (so-called “spring and parachute”, respectively) of a high drug concentration in the GI tract are crucial prerequisites for efficient oral absorption (i.e., Fick’s first law) [12]. In general, the drug amorphization approach guarantees a higher solubility than that of crystalline polymorphs due to supersaturation [13]. However, the thermodynamically unstable drug concentration eventually leads to precipitation, forming stable drug crystals again, which may exhibit low solubility and slow dissolution rate [12]. Thus, appropriate precipitation inhibitors (PIs) that can delay the drug crystallization need to be blended into the supersaturable formulations [14]. Polymers, such as Eudragits, hypromellose, and polyvinylpyrrolidones (PVPs), can inhibit precipitation by directly interfering with the nucleation or growth of drug crystals or by increasing the viscosity of media [12,15]. Non-ionic surfactants, such as Tween 20 and Cremophor RH40, can also serve as PIs by increasing the apparent drug solubility, forming micelles [14]. It is worth noting that Pluronics, non-ionic PEGylated polymers that possess both polymeric and surfactant-like properties, display the parachute effect even below their critical micelle concentrations because of their polymeric features [16]. 

The nanonization approach can enhance the dissolution rate of drugs (i.e., Noyes–Whitney equation), thereby accelerating their oral absorption [17]. Among various types of nanoscale carriers, liposomes and niosomes have shown various advantages as oral delivery systems for poorly water-soluble drugs [18,19]. Liposomes are vesicles of phospholipid bilayers of which fabrication methods are well established, encompassing mechanical dispersion (e.g., thin-film hydration, sonication or microfluidization), solvent dispersion (e.g., double emulsion or reverse-phase evaporation), and detergent removal (e.g., dialysis or dilution) methods [20,21]. Due to the lipid bilayer structure, hydrophobic drugs like CXB can be readily incorporated into the liposomes with a high loading efficiency [11]. In addition, the enhancing effect of phospholipids can improve drug permeability [2]. Niosomes possess vesicular structures like liposomes but are mainly composed of non-ionic surfactants [19,22]. Although the fabrication methods of niosomes are almost the same as those of liposomes [23], the difference in composition allows the higher chemical stability, more flexible storage conditions, and lower production [24,25]. However, niosomes are associated with physical stability problems, such as aggregation, fusion, precipitation, and drug leakage during storage [26]. Moreover, the abovementioned preparation methods are still complex, time-consuming, and less reproducible, which impedes their clinical translation [26,27].

Herein, using a simple lyophilization method, we designed CXB-loaded amorphous solid dispersions, namely, proliponiosomes (PLNs) that can generate lipid/non-ionic surfactant-based nanovesicles (i.e., liponiosomes) spontaneously when dissolved in aqueous media. PEGylated Eudragit L100 polymers were synthesized as novel micelle-forming polymeric surfactants, which were blended into PLNs as PIs, together with phospholipid and other small molecular non-ionic surfactants. The physical state of CXB in the PLNs was evaluated by assessing thermal behaviors and X-ray diffraction patterns. The generation of CXB-loaded liponiosomes was confirmed via microscopic observation and particle size measurement. The dissolution and permeability of the developed PLNs were characterized using dialysis and Caco-2 cell monolayer models, respectively. The in vivo pharmacokinetic properties and systemic toxicities were also investigated in rats.

## 2. Materials and Methods

### 2.1. Materials

CXB was bought from Tokyo Chemical Industry (Tokyo, Japan). Eudragit L100 (125 kDa) was donated by Evonik Industries AG (Essen, Germany). PEG 1000, amine-functionalized methoxy PEGs (mPEG-NH_2_ 2000 and 5000), *N*-hydroxysuccinimide (NHS), 1-ethyl-3-(3-dimethylaminopropyl) carbodiimide (EDC), trimethylamine (TEA), SDS, Solutol HS 15 (SHS), tween 80, 4-(2-hydroxyethyl)piperazine-1-ethanesulfonic acid (HEPES), Hank’s balanced salt solution (HBSS), sodium bicarbonate, and d-glucose were purchased from Sigma–Aldrich (St. Louis, MO, USA). Soy phosphatidylcholine (SPC; Lipoid S75, fat-free soybean phospholipids with 70% PC, Lipoid GmbH, Ludwigshafen, Germany) was gifted by Phytos (Anyang, Korea). Ethanol, dimethyl formamide (DMF), and dimethyl sulfoxide (DMSO) were supplied by Daejung Chemicals & Metals Co., Ltd. (Seoul, Korea). Dulbecco’s modified Eagle medium (DMEM), penicillin-streptomycin, and fetal bovine serum (FBS) were purchased from Gibco Life Technologies, Inc. (Carlsbad, CA, USA).

### 2.2. Synthesis and Characterization of Eudragit L100-PEGs

PEGylation of Eudragit L100 (EL) was conducted via EDC/NHS coupling reaction. Briefly, EDC (92 mg; 0.48 mmol) dissolved in DMF (4 mL) containing TEA (10 μL; 0.072 mmol) was added dropwise to EL (200 mg; 0.0016 mmol) solution in DMF (11 mL). NHS (18.4 mg; 0.16 mmol) solubilized in DMF (1 mL) was slowly added to the mixture and stirred for 30 min. The activated EL solution was mixed with mPEG-NH_2_ 2000 (256 mg; 0.128 mmol) or 5000 (640 mg; 0.128 mmol) dissolved in DMF (15 mL) and stirred for 24 h at 45 °C. The mixture was dialyzed against water for 2 days using a regenerated cellulose tubing (molecular weight cut-off [MWCO]: 6–8 kDa; CelluSep; Membrane Filtration Products, Seguin, TX, USA). The dialysis products were concentrated using a centrifugal filter (MWCO: 30 kDa; Vivaspin 20; Sartorius, Goettingen, Germany) at 3000× *g* for 30 min and reconstituted with 20 mL of double-deionized water (DDW). The resulting products, EL-PEG 2000 and EL-PEG 5000 (ELP_2k_ and ELP_5k_, respectively), were stored at −20 °C for further experiments.

The synthesis of ELP was confirmed using proton nuclear magnetic resonance (^1^H-NMR; JNIM-ECA 600; JEOL, Tokyo, Japan). All samples, including EL, PEG 1000, ELP_2k_, and ELP_5k_, were dissolved in DMSO-d_6_. The physical mixtures of EL and PEG 1000 were prepared with various weight ratios by blending the DMSO-d_6_ solutions. The PEG content of ELP was calculated by using the correlation between the weight ratios of physical mixtures and the integration ratios of representative NMR signals indicating PEG (3.5 ppm; ethylene groups) and EL (0.5–1.5 ppm; methyl and methylene groups). The calibration curve was constructed using linear regression.

### 2.3. Preparation and Characterization of CXB-Loaded Proliponiosomes (CXB@PLNs)

CXB-loaded proliponiosomes (CXB@PLNs) were prepared by a lyophilization method. In brief, CXB (5 mg), SHS (10 mg), tween 80 (10 mg), PEG 1000 (100 mg), and SPC (10 mg, solubilized in 10 μL of ethanol) were dissolved in DMSO (1 mL). ELP_2k_ or ELP_5k_ (15 mg) was added to the mixture, followed by vortex-mixing for 1 min and snap-freezing in liquid nitrogen. Lyophilization was conducted using a freeze-dryer (FDU-8603; Operon, Gimpo, Korea) at −90 °C (0.4 Pa) for 2 days. The resulting ELP_2k_- and ELP_5k_-containing CXB@PLNs (F1 and F2, respectively) were kept at −70 °C for further use. The blank F1 and F2 were prepared with the same method, except that CXB was not added.

The experimental protocols regarding the characterization of CXB@PLNs, including the surface morphology (by scanning electron microscopy [SEM]), physical state (by differential scanning calorimetry [DSC] and powder X-ray diffractometry [PXRD]), and CXB content (by reversed-phase high-performance liquid chromatography [RP-HPLC]), are presented in Supplementary Materials.

Liponiosomes (LNs) were generated by reconstituting F1 or F2 (150 mg) in DDW (1.5 mL) by vortex-mixing (10 s). The morphology of LNs was observed by using a transmission electron microscopy (TEM; LIBRA 120; Carl Zeiss AG). An aliquot (50 μL) of the dispersion was placed onto the surface of a 200-mesh carbon-coated copper grid and negatively stained with uranyl acetate (UA) solution (2%). The sample observation was conducted at an accelerating voltage of 80 kV. The average particle size with polydispersity index (PDI) and zeta potential values of the reconstituted LNs were measured by dynamic light scattering (DLS) and laser Doppler electrophoresis, respectively, using a Zetasizer Ultra (Malvern Instruments Ltd., Malvern, UK). The encapsulation efficiency (EE) of LNs was determined after removing unloaded CXB from the reconstituted LNs using a syringe filter (pore size: 0.45 μm; Minisart RC15; Sartorius). The CXB concentration in filtrate was quantified by the RP-HPLC method presented in Supplementary Materials (Section S1.1).

### 2.4. Release Test

The drug-releasing property of CXB@PLNs was evaluated by using a dialysis method. The formulations (CXB suspension, F1, and F2 containing 200 μg of CXB) were loaded into a regenerated cellulose tubing (MWCO: 2 kDa; CelluSep H1; Membrane Filtration Products). The tubes were immersed into release media (20 mL) of various pH conditions, including pH 1.2 (containing 40 mg of sodium chloride and 140 µL of 1 M hydrochloric acid solution), 4.5 (containing 24 mg of sodium acetate and 48 mg of acetic acid), 6.8 (containing 156 mg of sodium phosphate monobasic; adjusted with 1 M sodium hydroxide solution), and 7.5 (containing 156 mg of sodium phosphate monobasic; adjusted with 1 M sodium hydroxide solution), all of which were supplemented with SDS (0.4%, *w*/*v*) to maintain sink condition. CXB suspension was prepared by grinding drug powder with DDW containing 0.5% (*w*/*v*) sodium carboxymethylcellulose (NaCMC) using a sterilized mortar and pestle. The experiment was conducted in a shaking water bath at 37 °C (50 rpm). Aliquots (0.3 mL) of the buffers were collected at predetermined time points (0.5, 1, 2, 4, 6, 8, and 24 h), and an equal volume was replenished after each sampling. The collected samples were diluted with ACN and analyzed using the RP-HPLC method in Supplementary Materials (Section S1.1).

### 2.5. Cytotoxicity and Permeability Assays

Cytotoxicity of the synthesized polymers (ELP_2k_ and ELP_5k_) and CXB@PLNs was assessed in Caco-2 cells (Korean Cell Line Bank; Seoul, Korea) by 3-(4,5-dimethylthiazol-2-yl)-5-(3-carboxymethoxyphenyl)-2-(4-sulfophenyl)-2H-tetrazolium (MTS)-based assay. Caco-2 cells were cultured with DMEM containing FBS (10%, *v*/*v*), penicillin (100 U/mL), and streptomycin (100 μg/mL) in a 5% CO_2_ atmosphere and 95% relative humidity at 37 °C. The cells were seeded onto 96-well plates at a density of 1.0 × 10^4^ cells per well and incubated for 24 h. The cells were exposed to various concentrations of ELPs or CXB@PLNs (2, 5, 10, 20, 50, 100, 200, 500, 1000, and 2000 μg/mL; dispersed in culture media) for 24 or 48 h and subsequently incubated with MTS reagent (CellTiter 96 AQueous One Solution Cell Proliferation Assay Reagent; Promega Corp., Madison, WI, USA) for additional 4 h at 37 °C. The absorbance was measured at a wavelength of 490 nm by using a microplate reader (Multiskan GO; Thermo Scientific, Waltham, MA, USA).

The Caco-2 permeability assays were conducted using a previously reported method by our group [2]. More detailed information can be found in Supplementary Materials.

### 2.6. Pharmacokinetic Study and Blood Biochemistry Test

The pharmacokinetic study was performed in a Sprague-Dawley rat model previously reported by our group [2]. Further information regarding the animal and experimental protocol is presented in Supplementary Materials. Pharmacokinetic parameters, including area under the plasma concentration-time curve from time zero to 24 h (AUC), peak plasma concentration (C_max_), and time to reach C_max_ (T_max_), were calculated using non-compartmental analysis (WinNonlin, version 3.1, NCA 201; Pharsight, Mountain View, CA, USA). Relative bioavailability (%) was calculated by dividing the average AUC value of each formulation with that of CXB suspension.

The blood biochemistry test was also performed in Sprague-Dawley rats (body weight: 235 ± 5 g; Orient Bio). Blood samples were collected at 24 h after an oral administration of F1 or F2 (as CXB, 2 mg/kg) using the cannulation method presented in Supplementary Materials. The serum was separated by incubating blood samples at room temperature for 1 h and centrifuging at 16,000× *g* for 10 min. The levels of serum alanine transaminase (ALT), albumin (ALB), total protein (TP), blood urea nitrogen (BUN), and serum creatinine (SCr) were analyzed using Fuji Dri-Chem 3500s (Fujifilm Corp., Tokyo, Japan).

### 2.7. Statistical Analysis

All assays were conducted at least three times of which results were presented as the mean ± standard deviation (SD). The statistical analysis (one-way analysis of variance with Tukey’s multiple comparison test) was performed using an SPSS statistics software (Version 21.0; IBM Corp, Armonk, NY, USA).

## 3. Results and Discussion

### 3.1. Synthesis and Characterization of ELPs

**Figure 1:**
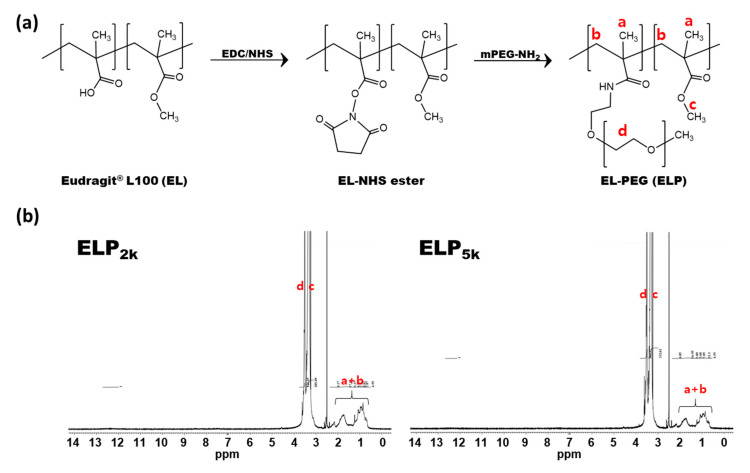
PEGylation of EL via EDC/NHS coupling reaction. Synthetic scheme (**a**) and ^1^H-NMR spectra (**b**) of ELP_2k_ and ELP_5k_ are presented (enlarged versions can be found in Supplementary Materials Figure S2). Chemical shifts are assigned to the corresponding hydrogen atoms (red font).

ELPs were synthesized via EDC/NHS coupling reaction, where the activated carboxylic acid group of EL was conjugated with the amine group of mPEG-NH_2_ (Figure 1a). Although it is well known that EDC/NHS reagents can form amide bonds at room temperature efficiently [28], ELPs were obtained only at the mildly heated condition. In our preliminary study, the reaction was also performed at room temperature only to result in precipitation during dialysis against DDW. However, ELPs prepared at 45 °C appeared to be translucent with no precipitation during the dialysis, which implies the successful conjugation of PEGs, considering the insolubility of EL in DDW.

The synthesis of ELPs was also confirmed using ^1^H-NMR analysis. As shown in Figure 1b and Supplementary Materials Figure S1, the representative signal of PEG (*d*; 3.5 ppm) can be observed in both ELP spectra, where the acidic proton signal of EL (12.4 ppm) also disappeared (Supplementary Materials Figure S2). The PEG content of ELPs was quantified by using ^1^H-NMR spectra of EL/PEG physical mixtures. The peak area ratio of representative EL and PEG signals (Y; methyl and methylene peaks of EL [*a* + *b*; 0.5–1.5 ppm] vs. ethylene peak of PEG [*d*; 3.5 ppm]) and corresponding weight ratio (X) were used to construct standard curve: Y = 5.226·X + 0.047 (*R*^2^ = 1.000). The calculated PEG contents of ELP_2k_ and ELP_5k_ were 36.6 ± 0.1% (*w*/*w*) and 59.5 ± 0.2% (*w*/*w*), respectively. The molar substitution ratios of PEG to EL were almost the same for ELP_2k_ and ELP_5k_, exhibiting 36.0 and 36.7, respectively, which could be attributed to the same molar feed ratios (i.e., approximately 80 PEGs per each EL chain). The substitution yields (%) were calculated to be 45.0% (= 36.0/80 × 100%) and 45.9% (= 36.7/80 × 100%) for ELP_2k_ and ELP_5k_, respectively. Degrees of substitution (%) were determined as the molar ratio of conjugated PEGs to total carboxylic acid groups in each EL chain (approximately 672), which give 5.36% (= 36.0/672 × 100%) and 5.46% (= 36.7/672 × 100%) for ELP_2k_ and ELP_5k_, respectively. To confirm the removal of unconjugated mPEG-NH_2_ from the products, ninhydrin staining was performed (Supplementary Materials Figure S3). The contents (%) of unconjugated mPEG-NH_2_ in ELP_2k_ and ELP_5k_ were estimated to be less than 1% and 2%, respectively.

### 3.2. Preparation and Characterization of CXB@PLNs in Solid State

**Figure 2:**
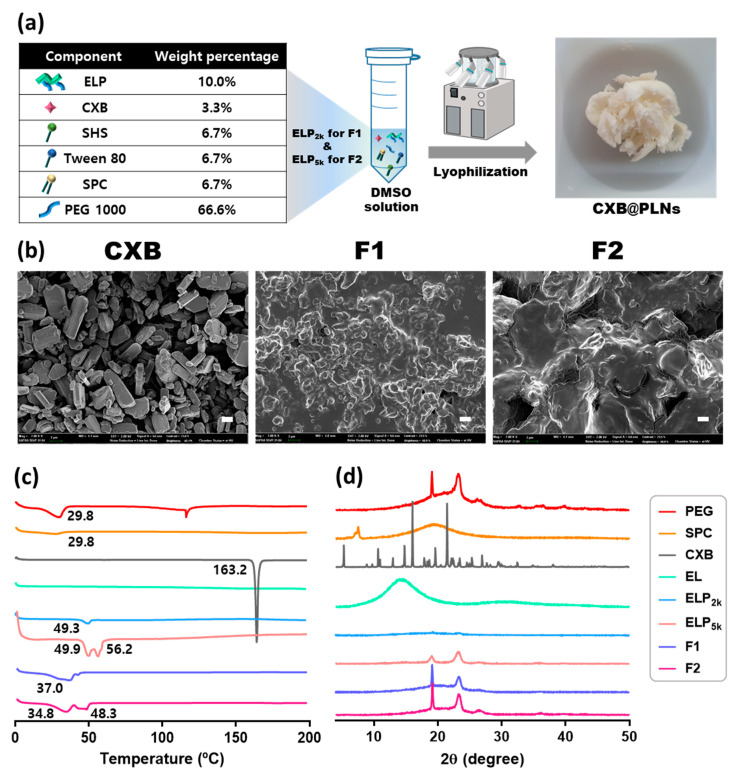
Preparation and solid-state characterization of CXB@PLNs. (**a**) Composition and visual appearance of CXB@PLNs are presented with the fabrication process. (**b**) SEM images of CXB, F1, and F2. The stacked plate-like morphology of CXB powder disappeared in F1 and F2. The length of the scale bar is 2 μm. (**c**) DSC thermograms and (**d**) PXRD diffractograms of ELPs, CXB, CXB@PLNs, and other excipients used are shown.

The composition and visual appearance of CXB@PLNs are presented in Figure 2a, which were fabricated by lyophilization of the DMSO solution of those ingredients. Compared to the theoretical CXB content, the prepared CXB@PLNs exhibited 88.6 ± 3.3% (F1) and 91.0 ± 4.5% (F2) of loading efficiency. SEM observation revealed that CXB powder displayed a stacked plate-like morphology, which is consistent with the appearance of a known CXB polymorph (Form III) (Figure 2b) [29]. Although ELP_2k_-containing CXB@PLNs (F1) displays a more granular surface than those containing ELP_5k_ (F2), neither exhibited the characteristic shape of CXB powder. These results imply that CXB was converted to the amorphous form (or other crystalline polymorphs) during the preparation process.

The physical state of CXB@PLNs was further investigated using DSC. The DSC thermograms of ELPs, CXB, CXB@PLNs, and other excipients used are shown in Figure 2c. PEG 1000 exhibited a broad melting peak at around 30 °C, which correlates well with the previous report [30]. SPC also showed a subtle endothermic depression at approximately 20–30 °C, which corresponds to its melting temperature [31]. EL displayed a flat baseline, indicating the absence of a distinct phase transition within the tested temperature range. However, both ELP_2k_ and ELP_5k_ showed depressions at around 50–60 °C. Considering that the melting temperatures of PEG 2000 and 5000 are 49–52 °C and 60–64 °C, respectively, those endothermic peaks can be explained by the phase transition of the PEG chains in ELPs. The depression patterns of F1 and F2 ranging over 30–50 °C appear to be the combined melting peaks of PEG 1000, SPC, and ELPs with their weight percentages taken into account. Meanwhile, CXB exhibited a sharp endothermic peak at 163 °C, which corresponds to the melting point of Form III polymorph [29]. Interestingly, this characteristic peak disappeared in the DSC curves of F1 and F2, which indicates that the amorphization of CXB occurred during the preparation of CXB@PLNs.

A more detailed investigation of the crystallinity of CXB@PLNs was conducted using PXRD (Figure 2d). PEG 1000 showed two distinctive diffraction peaks at 19.1° and 23.2°, which are derived from the (120) and (032) planes in Miller index notation, respectively [32]. SPC displayed a non-crystalline pattern, which is in good accordance with the gradual depression observed in DSC analysis. EL showed no sign of crystallinity, whereas ELP_5k_ displayed the characteristic peaks of PEG at 19.0° and 23.2°. This difference can be attributed to the introduced PEG chains in ELP_5k_. Although ELP_2k_ showed very subtle elevation at those 2θ values, the almost flat diffractogram indicates the non-crystalline property of ELP_2k_. As expected from SEM and DSC data, CXB exhibited a characteristic pattern of Form III polymorph, where sharp peaks at 5.2°, 10.6°, 13.0°, 14.8°, 16.1°, 19.7°, and 21.5° can be observed [29]. However, CXB@PLNs exhibited diffraction patterns similar to that of PEG 1000 with no characteristic signals of CXB. This result indicates the loss of crystallinity of CXB during the fabrication of F1 and F2, supporting the DSC results.

### 3.3. Characterization of LNs Generated by Reconstitution of CXB@PLNs

**Figure 3:**
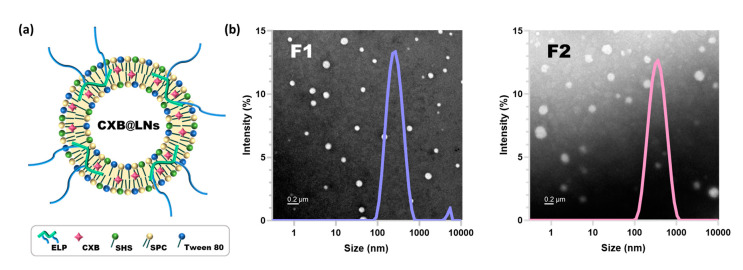
LNs generated by the reconstitution of CXB@PLNs. (**a**) Schematic illustration of CXB@LNs is displayed. (**b**) Size distribution diagrams of F1- and F2-LNs assessed by DLS analysis are presented with corresponding TEM images (background). The length of the scale bar is 200 nm.

CXB@PLNs were designed to be rapidly dispersed in the aqueous milieu, generating nanoscale LNs (Figure 3a). The phospholipid (SPC) and non-ionic surfactants (SHS and tween 80) were incorporated to CXB@PLNs, as they are known to form LNs together [33,34]. SPC is a mixture of diacyl-conjugated glycero-3-phosphocholines of which fatty acid chains mostly consisting of palmitate, stearate, oleate, linoleate, and linolenate [35]. SHS is a PEGylated fatty acid ester that has 15-PEG units conjugated to 12-hydroxystearate. Tween 80 possesses a structure of sorbitan monooleate decorated with 20-PEG units. Due to the amphiphilic structures, these three compounds can serve as not only drug solubilizers but also absorption enhancers [36]. ELPs were added to the PLNs as main PIs, as these polymers exhibited micelle-forming property. Our preliminary study revealed that ELP_2k_ and ELP_5k_ dispersions in DDW exhibited mean diameters of 49.4 ± 28.0 nm (PDI: 0.46 ± 0.18) and 70.2 ± 9.8 nm (PDI: 0.45 ± 0.10), respectively. PEG 1000 was blended to promote the rapid dispersion of CXB@PLNs in aqueous media. Theoretically, PEGs with lower molecular weight may achieve faster dispersion in aqueous conditions. In the preliminary study, however, the CXB@PLNs containing PEG 600 exhibited sticky and paste-like properties after lyophilization (data not shown).

Reconstitution of F1 and F2 in DDW produced LNs with mean diameters of 231.7 ± 1.5 nm and 292.0 ± 3.5 nm of which PDI values were 0.27 ± 0.01 and 0.25 ± 0.01, respectively (Figure 3b and Table 1). The PDI values less than 0.3 indicate that the LNs possess a narrow size distribution [37]. CXB@PLNs containing EL instead of ELPs were also prepared and evaluated based on DLS analysis only to find out that the resulting formulation exhibited incomplete reconstitution in DDW, thus discouraging further investigation (Supplementary Materials Figure S4). The generation of LNs was further confirmed by UA-staining assisted TEM imaging, where both F1- and F2-LNs displayed spherical morphology (Figure 3b). Interestingly, the mean diameters of F1- and F2-LNs estimated from the TEM images (42.0 ± 13.6 nm and 67.7 ± 14.3 nm, respectively) are in good accordance with the number-averaged particle sizes derived from DLS analysis (44.4 ± 4.7 nm and 70.0 ± 12.9 nm, respectively) (Supplementary Materials Figure S5). The zeta potential values of F1- and F2-LNs were 1.87 ± 0.13 mV and -5.41 ± 0.94 mV, respectively (Table 1). The EE of F1- and F2-LNs determined using 0.45 µm filters exhibited comparable values of 80.8 ± 0.3% and 89.2 ± 2.5%, respectively. Considering the reduced particle size and positively-charged zeta potential, F1-LNs could be more effective in the oral delivery of CXB than F2-LNs [38,39].

### 3.4. In Vitro CXB Release from CXB@PLNs

**Figure 4:**
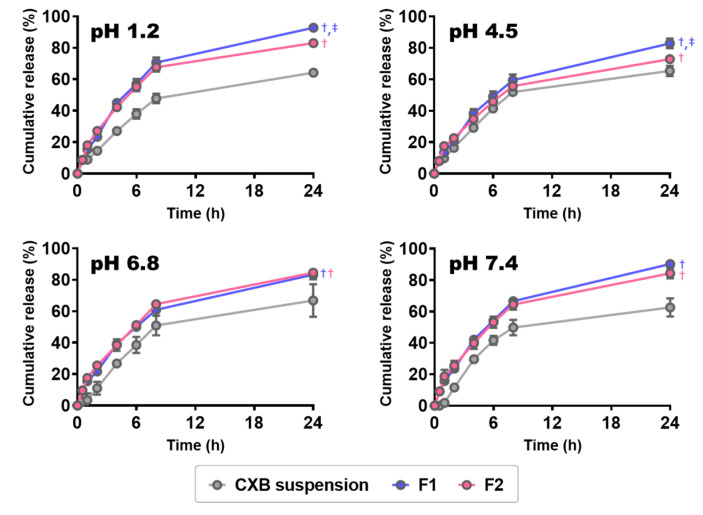
In vitro drug release profiles of CXB suspension, F1, and F2 at various pH values. Each point represents the mean ± SD (*n* = 3). ^†^ *p* < 0.05, compared to CXB suspension; ^‡^ *p* < 0.05, compared to F1.

The in vitro release test was conducted at various pHs of 1.2 (fasted-state stomach), 4.5 (fed-state stomach), 6.8 (proximal small intestine), and 7.4 (distal small intestine) [40]. The release profiles of CXB suspension, F1, and F2 are presented in Figure 4. The CXB suspension prepared by grinding CXB powder in NaCMC solution was employed as a control. It is worth noting that both F1 and F2 exhibited significantly higher cumulative release than CXB suspension in all tested pH conditions (*p* < 0.05). These results imply that the bottom-up processing that induces amorphization of CXB is more efficient in enhancing the dissolution rate than the top-down method that does not accompany a change in drug crystallinity (i.e., grinding). The amorphous form of CXB can provide an improved dissolution rate compared to its crystalline form because of weaker intermolecular forces [41]. Meanwhile, F1 showed comparable (at pH 6.8 and 7.4) or significantly higher (at pH 1.2 and 4.5) cumulative release than F2 (*p* < 0.05). As the absorption of CXB occurs throughout the GI tract [4], the enhancement in the dissolution rate at any physiologically-relevant pHs may improve the oral bioavailability of CXB, as suggested by previous reports [42,43,44].

### 3.5. Cytotoxicity of ELPs and Permeability of CXB@PLNs

**Figure 5:**
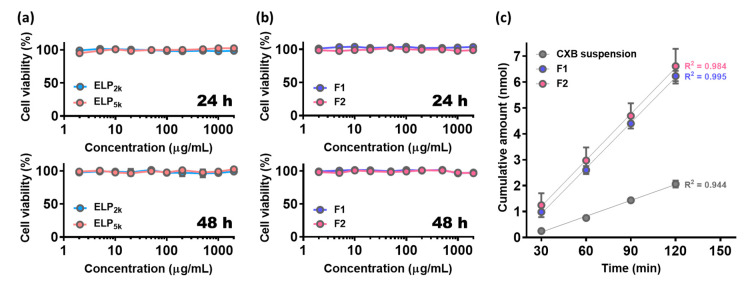
Cytotoxicity of ELPs (**a**) and CXB@PLNs (**b**) was evaluated in Caco-2 cells by MTS-based assay, where the cells were incubated with ELPs and CXB@PLNs at various concentrations (2–2000 μg/mL) for 24 and 48 h. Each point represents the mean ± SD (*n* = 5). (**c**) Cumulative amount of CXB transported across the Caco-2 cell monolayer according to incubation time. CXB suspension, F1, and F2 were administered to the A-side of Transwell insert. A simple linear regression was performed to calculate *P_app_* values. Data are presented as the mean ± SD (*n* = 5).

The cytotoxicity of ELPs and CXB@PLNs was evaluated using MTS-based assay, where the polymers and formulations were incubated with Caco-2 cells for 24 and 48 h. As shown in Figure 5a, the average cell viability was higher than 96.2% within the tested concentration range (2–2000 µg/mL), indicating negligible cytotoxicity of ELPs. F1 and F2 also exhibited no significant cytotoxicity in Caco-2 cells even after 48 h of incubation at the highest concentration (as cell viability, 97.6 ± 2.0% and 96.6 ± 3.1%, respectively) (Figure 5b), which can be explained by the non-cytotoxic and biocompatible nature of ELPs and all other excipients used [35,36,45,46].

Absorptive transport of CXB suspension, F1, and F2 was evaluated in the Caco-2 cell monolayer model (Figure S5b). Although F1 and F2 exhibited comparable apparent permeability (*P_app_*) values of 17.4 ± 0.5 and 17.7 ± 1.6 nm/sec, respectively, both of them were significantly different from *P_app_* of CXB suspension (6.1 ± 0.4 nm/sec) (*p* < 0.05). This could be attributed to the improved dissolution properties of CXB@PLNs (Section 3.4), which may have increased the dissolved CXB concentration in the apical side (A-side). In addition, the enhancing effects of phospholipid and non-ionic surfactants in CXB@PLNs may also have affected the increased permeability [36]. At the end of the transport experiment, trans-epithelial electrical resistance (TEER) values were measured to verify whether the increased permeability resulted from any damages to the cell monolayer. The average TEER values of CXB suspension, F1, and F2 at 120 min were 454 ± 72, 669 ± 262, and 643 ± 359 Ω·cm^2^, respectively, displaying no significant differences. This result suggests that F1 and F2 exhibited enhancement in permeability without causing serious damage to the monolayer compared to CXB suspension.

### 3.6. Pharmacokinetic Study and Blood Biochemistry Test

**Figure 6:**
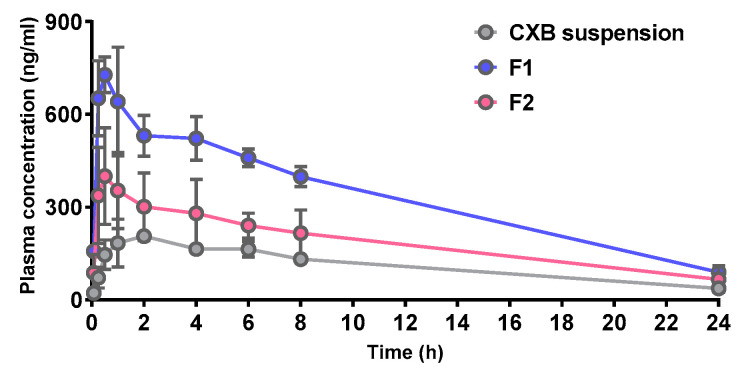
Plasma CXB concentration versus time profiles after an oral administration of CXB suspension, F1, and F2 at a dose of 2 mg/kg in rats. Data are presented as the mean ± SD (*n* = 4).

The pharmacokinetic properties of CXB@PLNs were evaluated in rats, where CXB suspension, F1, or F2 was orally administered at a CXB dose of 2 mg/kg. The plasma CXB concentration versus time profiles and pharmacokinetic parameters are presented in Figure 6 and Table 2, respectively. The AUC values of all groups were significantly different from among others (*p* < 0.05), showing rank order of CXB suspension (as relative bioavailability, 100%) < F2 (176%) < F1 (287%). Considering the low solubility of free CXB (≈1.23 ± 0.05 μg/mL at pH 6.8), the enhancement in oral absorption of CXB@PLNs can be mainly attributed to their improved dissolution properties [43]. Moreover, the generated LNs were designed to serve as both absorption enhancers and PIs in the GI tract, which may also have affected the increased oral bioavailability. A similar approach with CXB-loaded proliposomes composed of SPC, poloxamer 188, and sorbitol was reported by our group [2], where an improved dissolution of CXB was also translated to an enhanced oral absorption (1.73-fold, compared to free CXB). Meanwhile, the higher bioavailability of F1 than F2 can be explained by the difference in physicochemical properties (i.e., reduced mean diameter and positive surface charge) (Section 3.3), as well as the superior dissolution properties (Section 3.4). The average C_max_ values of F1 and F2 increased 3.45- and 1.83-fold compared to that of CXB suspension, which could also be explained by the improvement in dissolution behaviors. The T_max_ values of CXB suspension were highly variable, displaying a range of 1–6 h. However, F1 and F2 exhibited shorter values of 0.56 ± 0.31 and 0.63 ± 0.25 h, respectively. As these T_max_ values of CXB@PLNs are much faster than that of Celebrex (2.75 ± 1.50 h) [2], CXB@PLNs could be a promising CXB delivery platform for acute pain management, which requires rapid onset time [47].

Blood biochemistry tests were conducted in rats, where clinical biomarkers that are related to the damages to the liver (ALT, ALB, and TP) and kidney (BUN and SCr) were assessed after an oral administration of the developed formulations at a CXB dose of 2 mg/kg. As shown in Table 3, no significant difference was observed between the control (no treatment) and the formulation-treated groups, indicating that oral administration of CXB@PLNs at the administered dose caused no serious systemic toxicities. Together with the negligible cytotoxicity observed in Caco-2 cells (Section 3.5), this result supports the safety of CXB@PLNs as oral delivery platforms.

## 4. Conclusions

CXB@PLNs were prepared using a simple lyophilization method for the oral delivery of CXB, which contain ELPs, novel polymeric PIs, synthesized by the PEGylation of EL. The physical state of CXB@PLNs was investigated using DSC and PXRD analyses, which suggest that the amorphization of CXB occurred during the fabrication of PLNs. Generating LNs in aqueous conditions, CXB@PLNs displayed enhanced dissolution rate and permeability compared to CXB suspension. In vivo pharmacokinetic studies demonstrated the higher oral bioavailability and faster T_max_ of CXB@PLNs than those of CXB suspension. The blood biochemistry tests performed in rats confirmed no severe systemic toxicities of CXB@PLNs. Taken together, the developed PLNs could be promising platforms for improving the oral bioavailability of poorly water-soluble drugs, such as CXB.

## Figures and Tables

**Table 1:** Characterization of LNs generated from CXB@PLNs.

Formulation	Mean Diameter	Polydispersity Index	Zeta Potential	Encapsulation Efficiency
F1	231.7 ± 1.5 nm	0.27 ± 0.01	1.87 ± 0.13 mV	80.8 ± 0.3%
F2	292.0 ± 3.5 nm	0.25 ± 0.01	−5.41 ± 0.94 mV	89.2 ± 2.5%

Data are presented as mean ± SD (*n* = 3).

**Table 2:** Pharmacokinetic parameters after an oral administration of CXB suspension, F1, and F2 at a dose of 2 mg/kg in rats.

Parameter ^1^	CXB Suspension	F1	F2
AUC (μg∙h/mL)	2.66 ± 0.10	7.98 ± 0.64 ^†,‡^	4.46 ± 1.41 ^†^
C_max_ (ng/mL)	222 ± 27	765 ± 72 ^†,‡^	407 ± 149
T_max_ (h)	2.50 ± 2.30	0.56 ± 0.31	0.63 ± 0.25
Relative bioavailability (%)	100	300	168

^1^ Data are presented as the mean ± SD (*n* = 4); ^†^ *p* < 0.05, compared to CXB suspension group; ^‡^ *p* < 0.05, compared to F2 group.

**Table 3:** Blood biochemistry parameters after an oral administration of CXB@PLNs in rats.

Parameter ^1^	No Treatment	F1	F2
ALT (U/L)	126.5 ± 76.3	163.3 ± 53.8	147.0 ± 24.5
ALB (g/dL)	2.7 ± 0.3	2.4 ± 0.4	2.6 ± 0.3
TP (g/dL)	4.2 ± 0.5	4.3 ± 0.3	4.5 ± 0.3
BUN (mg/dL)	23.7 ± 9.3	24.0 ± 12.9	28.9 ± 1.3
SCr (mg/dL)	0.20 ± 0.01	0.17 ± 0.03	0.18 ± 0.02

^1^ Data are presented as the mean ± SD (*n* = 4).

## Supplementary Materials

The following are available online at https://www.mdpi.com/1999-4923/12/8/718/s1. Figure S1: ^1^H-NMR spectra of ELP_2k_ and ELP_5k_ in DMSO-d_6_ (~4.0 ppm); Figure S2: ^1^H-NMR spectrum of EL in DMSO-d_6_; Figure S3: Ninhydrin staining to confirm the removal of remaining mPEG-NH_2_ from ELPs; Figure S4: DLS analysis of LNs generated from EL-containing CXB@PLNs; Figure S5: Number-averaged diameters of LNs measured by TEM imaging and DLS analysis. Supplementary methods and a list of abbreviations are also available.

## References

[1] McCormack P.L. (2011). Celecoxib. Drugs.

[2] Jeon D., Kim K.-T., Baek M.-J., Kim D.H., Lee J.-Y., Kim D.-D. (2019). Preparation and evaluation of celecoxib-loaded proliposomes with high lipid content. Eur. J. Pharm. Biopharm..

[3] Frampton J.E., Keating G.M. (2007). Celecoxib: A review of its use in the management of arthritis and acute pain. Drugs.

[4] Paulson S.K., Vaughn M.B., Jessen S.M., Lawal Y., Gresk C.J., Yan B., Maziasz T.J., Cook C.S., Karim A. (2001). Pharmacokinetics of celecoxib after oral administration in dogs and humans: Effect of food and site of absorption. J. Pharmacol. Exp. Ther..

[5] Hammouda B. (2013). Temperature Effect on the Nanostructure of SDS Micelles in Water. J. Res. Natl. Inst. Stand. Technol..

[6] Kwon H.J., Heo E.-J., Kim Y.-H., Kim S., Hwang Y.-H., Byun J.-M., Cheon S.H., Park S.Y., Kim D.Y., Cho K.H. (2019). Development and evaluation of poorly water-soluble celecoxib as solid dispersions containing nonionic surfactants using fluidized-bed granulation. Pharmaceutics.

[7] Andrews G.P., Abu-Diak O., Kusmanto F., Hornsby P., Hui Z., Jones D.S. (2010). Physicochemical characterization and drug-release properties of celecoxib hot-melt extruded glass solutions. J. Pharm. Pharmacol..

[8] Lee J.H., Kim M.J., Yoon H., Shim C.R., Ko H.A., Cho S.A., Lee D., Khang G. (2013). Enhanced dissolution rate of celecoxib using PVP and/or HPMC-based solid dispersions prepared by spray drying method. J. Pharm. Investig..

[9] Morgen M., Bloom C., Beyerinck R., Bello A., Song W., Wilkinson K., Steenwyk R., Shamblin S. (2012). Polymeric nanoparticles for increased oral bioavailability and rapid absorption using celecoxib as a model of a low-solubility, high-permeability drug. Pharm. Res..

[10] Kishore N., Raja M.D., Kumar C.S., Dhanalekshmi U., Srinivasan R. (2016). Lipid carriers for delivery of celecoxib: In vitro, in vivo assessment of nanomedicine in rheumatoid arthritis. Eur. J. Lipid Sci. Technol..

[11] Deniz A., Sade A., Severcan F., Keskin D., Tezcaner A., Banerjee S. (2010). Celecoxib-loaded liposomes: Effect of cholesterol on encapsulation and in vitro release characteristics. Biosci. Rep..

[12] Guzmán H.R., Tawa M., Zhang Z., Ratanabanangkoon P., Shaw P., Gardner C.R., Chen H., Moreau J.P., Almarsson O., Remenar J.F. (2007). Combined use of crystalline salt forms and precipitation inhibitors to improve oral absorption of celecoxib from solid oral formulations. J. Pharm. Sci..

[13] Brouwers J., Brewster M.E., Augustijns P. (2009). Supersaturating drug delivery systems: The answer to solubility-limited oral bioavailability?. J. Pharm. Sci..

[14] Xu S., Dai W.-G. (2013). Drug precipitation inhibitors in supersaturable formulations. Int. J. Pharm..

[15] Li S., Pollock-Dove C., Dong L.C., Chen J., Creasey A.A., Dai W.G. (2012). Enhanced bioavailability of a poorly water-soluble weakly basic compound using a combination approach of solubilization agents and precipitation inhibitors: A case study. Mol. Pharm..

[16] Dai W.-G., Dong L.C., Li S., Deng Z. (2008). Combination of Pluronic/Vitamin E TPGS as a potential inhibitor of drug precipitation. Int. J. Pharm..

[17] Hattori Y., Haruna Y., Otsuka M. (2013). Dissolution process analysis using model-free Noyes–Whitney integral equation. Colloids Surf. B Biointerfaces.

[18] He H., Lu Y., Qi J., Zhu Q., Chen Z., Wu W. (2019). Adapting liposomes for oral drug delivery. Acta Pharm. Sin. B.

[19] Rajera R., Nagpal K., Singh S.K., Mishra D.N. (2011). Niosomes: A controlled and novel drug delivery system. Biol. Pharm. Bull..

[20] Brandelli A., Pinilla C.M.B., Lopes N.A., Rai M., Alves dos Santos C. (2017). Nanoliposomes as a Platform for Delivery of Antimicrobials. Nanotechnology Applied To Pharmaceutical Technology.

[21] Zeb A., Arif S.T., Malik M., Shah F.A., Din F.U., Qureshi O.S., Lee E.-S., Lee G.-Y., Kim J.-K. (2019). Potential of nanoparticulate carriers for improved drug delivery via skin. J. Pharm. Investig..

[22] Asgharkhani E., Fathi Azarbayjani A., Irani S., Chiani M., Saffari Z., Norouzian D., Akbarzadeh A., Atyabi S.M. (2018). Artemisinin-loaded niosome and pegylated niosome: Physico-chemical characterization and effects on MCF-7 cell proliferation. J. Pharm. Investig..

[23] Chen S., Hanning S., Falconer J., Locke M., Wen J. (2019). Recent advances in non-ionic surfactant vesicles (niosomes): Fabrication, characterization, pharmaceutical and cosmetic applications. Eur. J. Pharm. Biopharm..

[24] Ge X., Wei M., He S., Yuan W.E. (2019). Advances of Non-Ionic Surfactant Vesicles (Niosomes) and Their Application in Drug Delivery. Pharmaceutics.

[25] Ramadan A.A., Eladawy S.A., El-Enin A.S.M.A., Hussein Z.M. (2020). Development and investigation of timolol maleate niosomal formulations for the treatment of glaucoma. J. Pharm. Investig..

[26] Mittal S., Chaudhary A., Chaudhary A., Kumar A. (2020). Proniosomes: The effective and efficient drug-carrier system. Ther. Deliv..

[27] Yan-yu X., Yun-mei S., Zhi-peng C., Qi-neng P. (2006). Preparation of silymarin proliposome: A new way to increase oral bioavailability of silymarin in beagle dogs. Int. J. Pharm..

[28] Yan Q., Zheng H.-N., Jiang C., Li K., Xiao S.-J. (2015). EDC/NHS activation mechanism of polymethacrylic acid: Anhydride versus NHS-ester. RSC Adv..

[29] Lu G.W., Hawley M., Smith M., Geiger B.M., Pfund W. (2006). Characterization of a novel polymorphic form of celecoxib. J. Pharm. Sci..

[30] Hopp B., Smausz T., Tombácz E., Wittmann T., Ignácz F. (2000). Solid state and liquid ablation of polyethylene-glycol 1000: Temperature dependence. Opt. Commun..

[31] Drazenovic J., Wang H., Roth K., Zhang J., Ahmed S., Chen Y., Bothun G., Wunder S.L. (2015). Effect of lamellarity and size on calorimetric phase transitions in single component phosphatidylcholine vesicles. Biochim. Biophys. Acta Biomembr..

[32] Xiang H., Wang S., Wang R., Zhou Z., Peng C., Zhu M. (2013). Synthesis and characterization of an environmentally friendly PHBV/PEG copolymer network as a phase change material. Sci. China Chem..

[33] Mohammadi M., Haghirosadat B.F., Larypoor M., Ehsani R., Yazdian F., Rashedi H., Jahanizadeh S., Rahmani A. (2020). Synthesis, Characterization and Evaluation of Liponiosome Containing Ginger Extract as a New Strategy for Potent Antifungal Formulation. J. Clust. Sci..

[34] Naderinezhad S., Amoabediny G., Haghiralsadat F. (2017). Co-delivery of hydrophilic and hydrophobic anticancer drugs using biocompatible pH-sensitive lipid-based nano-carriers for multidrug-resistant cancers. RSC Adv..

[35] Scholfield C.R. (1981). Composition of soybean lecithin. J. Am. Oil Chem.’ Soc..

[36] Williams A.C., Barry B.W. (2012). Penetration enhancers. Advanced Drug Delivery Reviews.

[37] Danaei M., Dehghankhold M., Ataei S., Hasanzadeh Davarani F., Javanmard R., Dokhani A., Khorasani S., Mozafari M.R. (2018). Impact of Particle Size and Polydispersity Index on the Clinical Applications of Lipidic Nanocarrier Systems. Pharmaceutics.

[38] Du X.-J., Wang J.-L., Iqbal S., Li H.-J., Cao Z.-T., Wang Y.-C., Du J.-Z., Wang J. (2018). The effect of surface charge on oral absorption of polymeric nanoparticles. Biomater. Sci..

[39] Banerjee A., Qi J., Gogoi R., Wong J., Mitragotri S. (2016). Role of nanoparticle size, shape and surface chemistry in oral drug delivery. J. Control. Release.

[40] Khutoryanskiy V.V. (2015). Longer and safer gastric residence. Nat. Mater..

[41] Zhao P., Jiang H., Jiang T., Zhi Z., Wu C., Sun C., Zhang J., Wang S. (2012). Inclusion of celecoxib into fibrous ordered mesoporous carbon for enhanced oral bioavailability and reduced gastric irritancy. Eur. J. Pharm. Sci..

[42] Bohr A., Kristensen J., Stride E., Dyas M., Edirisinghe M. (2011). Preparation of microspheres containing low solubility drug compound by electrohydrodynamic spraying. Int. J. Pharm..

[43] Chawla G., Gupta P., Thilagavathi R., Chakraborti A.K., Bansal A.K. (2003). Characterization of solid-state forms of celecoxib. Eur. J. Pharm. Sci..

[44] Nasr M. (2010). In vitro and in vivo evaluation of proniosomes containing celecoxib for oral administration. AAPS PharmSciTech.

[45] Liu G., Li Y., Yang L., Wei Y., Wang X., Wang Z., Tao L. (2017). Cytotoxicity study of polyethylene glycol derivatives. RSC Adv..

[46] Mohammadzadeh R., Baradaran B., Valizadeh H., Yousefi B., Zakeri-Milani P. (2014). Reduced ABCB1 Expression and Activity in the Presence of Acrylic Copolymers. Adv. Pharm. Bull..

[47] Battisti W.P., Katz N.P., Weaver A.L., Matsumoto A.K., Kivitz A.J., Polis A.B., Geba G.P. (2004). Pain management in osteoarthritis: A focus on onset of efficacy—A comparison of rofecoxib, celecoxib, acetaminophen, and nabumetone across four clinical trials. J. Pain.

