# Supplementary material for: Preparation and Evaluation of Eudragit L100-PEG Proliponiosomes for Enhanced Oral Delivery of Celecoxib
Source: Pharmaceutics. 2020 Jul 30;12(8):718. doi: 10.3390/pharmaceutics12080718 (PMC7465340; doi:10.3390/pharmaceutics12080718)
Supplement: Supplementary file 1 [file pharmaceutics-12-00718-s001.pdf]

pharmaceutics

Supplementary Materials: Preparation and Evaluation of Eudragit L100-PEG Proliponiosomes for Enhanced Oral Delivery of Celecoxib

Min-Hwan Kim, Dong Hyun Kim, Duy-Thuc Nguyen, Han Sol Lee, Nae-Won Kang, Min-Jun Baek, Jiseon An, So-Yeol Yoo, Yong-Hyeon Mun, Wonhwa Lee, Ki-Taek Kim, Cheong-Weon Cho, Jae-Young Lee and Dae-Duk Kim

S1. Supplementary Methods

S1.1. Characterization of CXB@PLNs

The surface morphology of CXB@PLNs was observed using a scanning electron microscopy (SEM; SUPRA 55VP; Carl Zeiss AG, Oberkochen, Germany) at an accelerating voltage of 2.00 kV. CXB, F1, and F2 were placed onto carbon taped stub and sputter-coated with platinum under vacuum for 240 s (sputter current: −30 mA). The physical state of CXB loaded in PLNs was evaluated using differential scanning calorimetry (DSC; Discovery DSC; TA Instrument, New Castle, DE, USA). Samples (CXB, PEG 1000, SPC, EL, ELP_{2k}, ELP_{5k}, F1, and F2) were sealed in aluminum pans, and the heat flow was scanned from 0 to 200 °C (heating rate: 10 °C/min). The crystallinity of CXB@PLNs was assessed using a powder X-ray diffractometry (PXRD; D8 ADVANCE; Bruker, Billerica, MA, USA) equipped with Cu $K\alpha_1$ radiation (1.5418 Å). Samples (CXB, PEG 1000, SPC, EL, ELP_{2k}, ELP_{5k}, F1, and F2) were scanned over a 2θ range of 4–50° with a step angle of 0.02° (scan speed: 0.5 sec/step).

The CXB content (%) of F1 and F2 was measured using a reversed-phase high-performance liquid chromatography (RP-HPLC) system (Agilent 1260 infinity; Agilent Technologies, Palo Alto, CA, USA) equipped with a Kinetex C18 column (4.6 mm × 250 mm, 5 μm; Phenomenex, Torrance, CA, USA) and a C18 guard column (2.0 × 4 mm; Phenomenex). The mobile phase consisted of acetonitrile (ACN) with 0.1% TEA and phosphate buffer (10 mM; pH 9.0) with 0.1% TEA (70:30, *v*/*v*). The detection wavelength and flow rate were 260 nm and 1.0 mL/min, respectively. The standard curve constructed using the relationship between the peak area (Y) and CXB concentration (X) is Y = 27.06·X + 0.01337 (R^2 = 1.000). The analytical samples were prepared by disrupting CXB@PLNs in DMSO at a concentration of 100 mg/mL and diluting the mixture 100-fold with the mobile phase. The injection volume and the lowest CXB concentration of the standard curve were 10 μL and 500 ng/mL, respectively. The retention time and total run time were 4.1 and 10.0 min, respectively.

S1.2. Caco-2 Permeability Assay

Caco-2 cells were cultured under the abovementioned condition and trypsinized at 70–80% confluency. The harvested cells were seeded onto Transwell-COL (growth area: 1.12 cm^2; pore size: 0.4 μm; Corning Inc., Corning, NY, USA) at 3.0 × 10^5 cells per an insert. Absorptive transport of the developed formulations was evaluated after the trans-epithelial electrical resistance (TEER) values of Caco-2 cell monolayers reach over 500 Ω·cm^2. The cell monolayers were pre-incubated with transport medium (TM; HBSS with 25 mM of HEPES, 0.35 g/L of sodium bicarbonate, and 1.95 g/L of D-glucose) at 37 °C for 30 min. F1 or F2 dispersed in TM (500 μL; as CXB, 500 μM) was administered to the apical side (A-side) of the insert. CXB suspension in 0.5% (*w*/*v*) NaCMC-containing TM (control group; 500 μL; as CXB, 500 μM) was prepared by using a sterilized mortar and pestle. The insert was placed into a well containing 1.5 mL of fresh TM (basolateral side (B-side)) and incubated in a shaking water bath at 37 °C (50 rpm). The insert was moved to another well containing 1.5 mL of fresh TM every 30 min (total incubation time: 2 h). The B-side samples were collected, and the transported amount of CXB was analyzed using a previously reported HPLC-tandem mass spectrometry (LC-MS/MS) method [2]. The chromatographic separation of CXB and valsartan (VST; internal standard) was performed on an Agilent 1260 Infinity HPLC system (Agilent Technologies) equipped with a Poroshell 120 EC-C18 column (4.6 × 50 mm, 2.7 μm; Agilent Technologies). The mobile phase consisted of ACN and 5

mM ammonium formate buffer (90:10, *v*/*v*), and the flow rate was 0.4 mL/min. The analytical samples were prepared by diluting an aliquot (50 μL) of B-side sample with ACN (450 μL) containing VST (100 ng/mL), and the injection volume was 5 μL. Mass spectrometric detection was achieved using an Agilent 6430 Triple Quad LC/MS system (Agilent Technologies) with negative electrospray ionization (ESI) mode. The mass transitions from precursor to product ion were *m*/*z* 380.2 → 316.2 for CXB and *m*/*z* 434.1 → 350.0 for VST. The fragmentor voltage/collision energy values were 170 V/19 eV for CXB and 140 V/18 eV for VST. The data acquisition and processing were performed using MassHunter Workstation Software Quantitative Analysis (Version B.05.00; Agilent Technologies). The retention times of CXB and VST were 1.7 and 1.3 min, respectively, and the total run time was 3 min. The lower limit of quantitation (LLOQ) was determined as 5 ng/mL. The apparent permeability (P_{app}) was calculated using the below equation (1):

$$P_{app}\,(\text{nm/sec}) = \frac{\delta Q}{\delta t}\cdot\frac{1}{A\cdot C_0} \quad (1)$$

where $\delta Q/\delta t$ is the transported amount of CXB per unit time, A is the surface area of cell monolayer, and C_0 is the initial CXB concentration of the A-side.

S1.3. Pharmacokinetic Study

The pharmacokinetic properties of CXB@PLNs were evaluated in male Sprague-Dawley rats (body weight: 235 ± 5 g; Orient Bio, Sungnam, Korea). The rats were acclimatized in Animal Center for Pharmaceutical Research (College of Pharmacy, Seoul National University, Seoul, Korea) at a temperature of 22 ± 2 °C and relative humidity of 55 ± 5%. Protocols for the pharmacokinetic study were approved by Animal Care and Use Committee of the College of Pharmacy, Seoul National University (approval number: SNU-190527-3). Rats were anesthetized with Zoletil 50 (Virbac, Carros, France) at a dose of 50 mg/kg *via* intramuscular injection. The left femoral artery was cannulated with polyethylene tubing (PE-50; Becton Dickinson Diagnostics, Sparks Glencoe, MD, USA), and CXB suspension (containing 0.5% [*w*/*v*] NaCMC), F1, or F2 was administered orally at a CXB dose of 2 mg/kg. Blood samples (≈150 μL) were collected from the femoral artery at 5, 15, 30, 60, 120, 240, 360, 480, and 1440 min after the administration and immediately centrifuged at 16,000 × *g* for 3 min at 4 °C. An aliquot (50 μL) of the supernatant (plasma) was deproteinized by vortex-mixing with ACN (200 μL; containing 100 ng/mL of VST as internal standard) for 5 min and centrifuging at 16,000 × *g* for 5 min. The CXB concentration of supernatants (analytical samples) was analyzed using the same LC-MS/MS method in Section 2.5, except that the mobile phase composition was switched to ACN and 5 mM ammonium formate buffer (95:5, *v*/*v*) for more efficient separation.

S2. Supplementary Figures

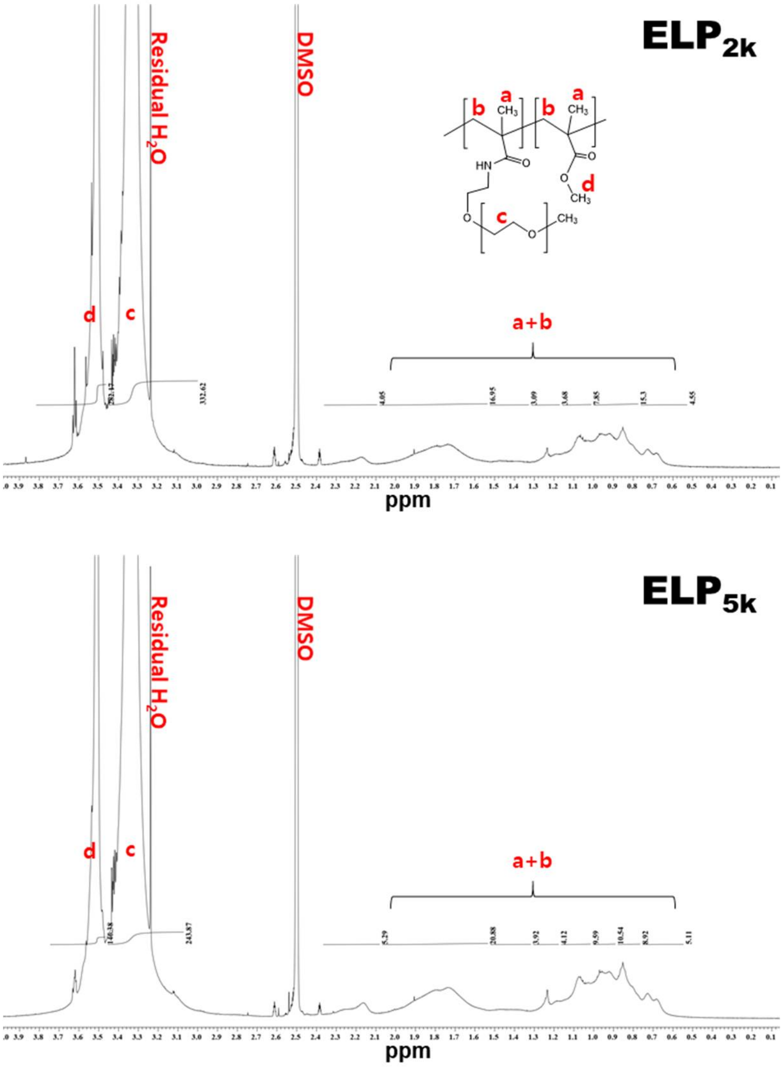

Figure S1. ^{1}H-NMR spectra of ELP_{2k} and ELP_{5k} in DMSO-d_6 (~4.0 ppm).

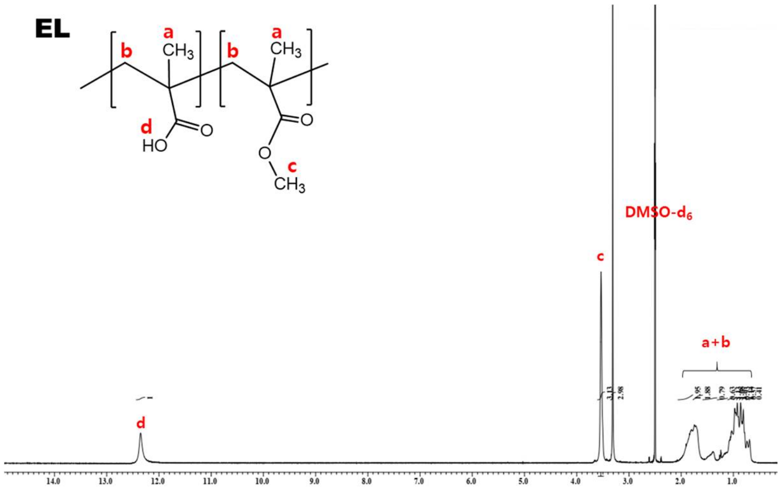

Figure S2. ^{1}H-NMR spectrum of EL in DMSO-d_6.

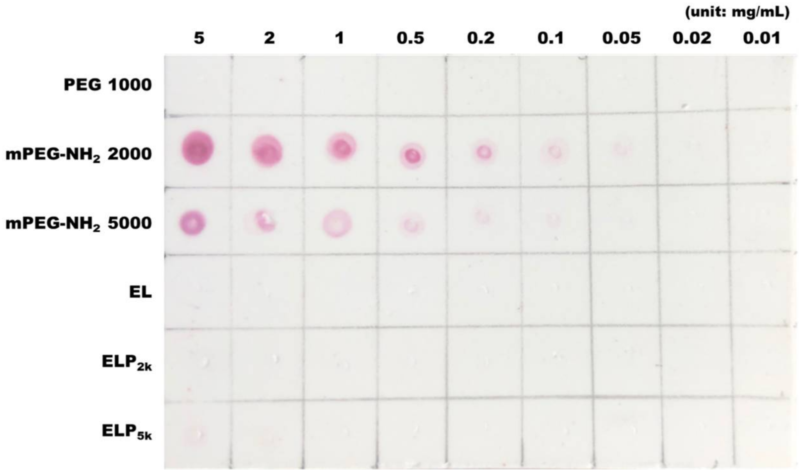

Figure S3. The removal of remaining $mPEG-NH_2$ from ELPs was confirmed using ninhydrin staining, which exhibits purple color in the presence of primary amine. Aliquots (0.75 μL) of ELP_{2k}, ELP_{5k}, $mPEG-NH_2$ 2000, and $mPEG-NH_2$ 5000 were spotted on a silica gel-coated plate at various concetrations (5, 2, 1, 0.5, 0.2, 0.1, 0.05, 0.02, and 0.01 mg/mL; dissolved in ethanol). After drying, the plate was soaked in a ninhydrin solution (3%, *w*/*v*) and placed on a hot-plate (120 °C) for 1 min. The color development was assessed visually, by which the remaining amounts of $mPEG-NH_2$ in ELPs were estimated. As negative controls, EL and PEG 1000 were also evaluated at the same concentrations. Lower limit of detection (LOD) concentrations of $mPEG-NH_2$ 2000 and 5000 were found to be 0.05 and 0.1 mg/mL, respectively. Considering that ELPs displayed no (ELP_{2k}) or very subtle (ELP_{5k}) color development at the highest concentration (5 mg/mL), the contents (%) of unconjugated $mPEG-NH_2$ in ELP_{2k} and ELP_{5k} can be estimated to be less than 1% (=0.05/5×100%) and 2% (=0.1/5×100%), respectively.

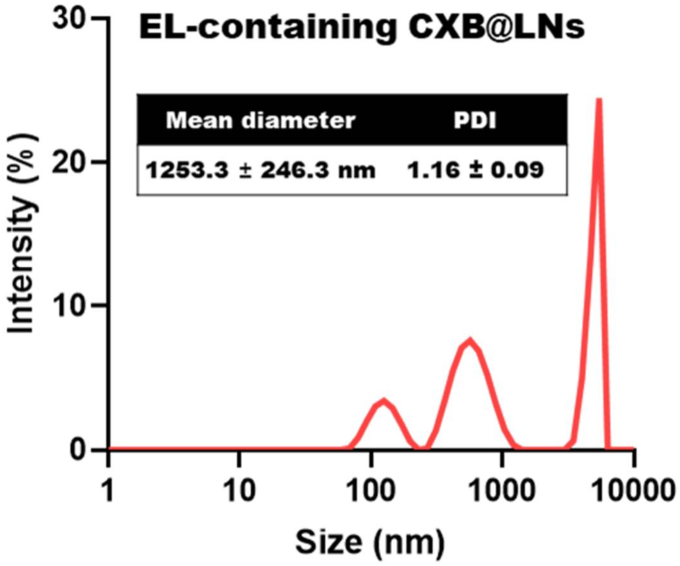

Figure S4. DLS analysis of LNs generated from EL-containing CXB@PLNs. The formulation was prepared using the same method in Section 2.3, except that the same amount of EL (15 mg) was added instead of ELPs. EL-containing CXB@PLNs exhibited an incomplete reconstitution in DDW even after 1 min of probe sonication at 20% amplitude (VC505; Sonics & Materials Inc., Newtown, CT, USA). The resulting suspension exhibited poor physicochemical properties, displaying mean diameters higher than 1 μm with broad size distribution (PDI of 1.16 ± 0.09).

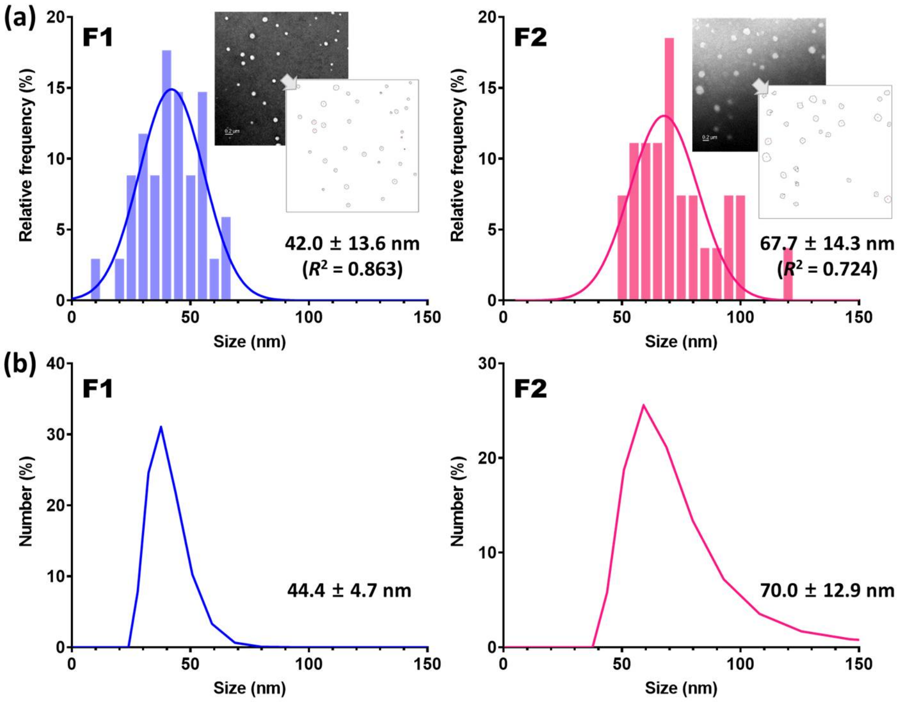

Figure S5. Number-averaged diameters of LNs measured by TEM imaging and DLS analysis. (**a**) TEM images (Figure 3b) were processed using ImageJ software (NIH Image, National Institutes of Health, Bethesda, MD, USA) (inset), where the sizes of all particles in the images were measured. The frequency distribution of particle size was plotted (bar chart) and fitted using a Gaussian distribution model (solid line), from which the average particle sizes of F1- and F2-LNs were estimated to be 42.0 ± 13.6 nm and 67.7 ± 14.3 nm, respectively. (**b**) Number-weighted size distributions of F1- and F2-LNs were converted from intensity-weighted ones in Figure 3b, displaying the number-averaged particle sizes of 44.4 ± 4.7 nm and 70.0 ± 12.9 nm, respectively.

S3. Abbreviations

Abbreviation	Full form	Abbreviation	Full form
^{1}H-NMR	proton nuclear magnetic resonance	LC-MS/MS	liquid chromatography-tandem mass spectrometry
ACN	acetonitrile	LLOQ	lower limit of quantitation
ALB	albumin	LNs	liponiosomes
ALT	serum alanine transaminase	MTS	3-(4,5-dimethylthiazol-2-yl)-5-(3-carboxymethoxyphenyl)-2-(4-sulfophenyl)-2*H*-tetrazolium
A-side	apical side	NaCMC	sodium carboxymethylcellulose
AUC	area under the plasma concentration-time curve from time zero to 24 h	NHS	*N*-hydroxysuccinimide
B-side	basolateral side	NSAID	non-steroidal anti-inflammatory drug
BUN	blood urea nitrogen	P_{app}	apparent permeability
C_{max}	peak plasma concentration	PDI	polydispersity index
COX	cyclooxygenase	PIs	precipitation inhibitors
CXB	celecoxib	PLNs	proliponiosomes
CXB@LNs	CXB-loaded liponiosomes	PXRD	powder X-ray diffractometry
CXB@PLNs	CXB-loaded proliponiosomes	RP-HPLC	reversed-phase high-performance liquid chromatography
DLS	dynamic light scattering	SCr	serum creatinine
DMEM	Dulbecco's modified Eagle medium	SD	standard deviation
DMF	dimethyl formamide	SDS	sodium dodecyl sulfate
DMSO	dimethyl sulfoxide	SEM	scanning electron microscopy
DS	degree of substitution	SHS	Solutol HS 15
DSC	differential scanning calorimetry	SPC	soy phosphatidylcholine
EDC	1-ethyl-3-(3-dimethylaminopropyl) carbodiimide	TEA	trimethylamine
EL	Eudragit L100	TEER	trans-epithelial electrical resistance
ELP_{2k}	EL-PEG 2000	TEM	transmission electron microscopy
ELP_{5k}	EL-PEG 5000	TM	transport medium
ESI	electrospray ionization	T_{max}	time to reach C_{max}
FBS	fetal bovine serum	TP	total protein
GI	gastrointestinal	UA	uranyl acetate
HBSS	Hank's balanced salt solution	VST	valsartan
HEPES	4-(2-hydroxyethyl)piperazine-1-ethanesulfonic acid		
